# An Ultra-Wide Range D-Shaped Fiber SPR Sensor with a Nanostructure of Gold–MoS_2_ and Sodium for the Simultaneous Measurement of Refractive Index and Temperature

**DOI:** 10.3390/s25020377

**Published:** 2025-01-10

**Authors:** Xinglian Lu, Xiantong Yu, Jun Zhou, Min Chang, Dunke Lu

**Affiliations:** 1 Shanghai Key Laboratory of Modern Optical System, School of Optical-Electrical and Computer Engineering, University of Shanghai for Science and Technology, Shanghai 200093, China; xtyu@usst.edu.cn (X.Y.); junzhou@usst.edu.cn (J.Z.); changmin@usst.edu.cn (M.C.); 2 School of Physics and Electronic Engineering, Guangzhou University, Guangzhou 510006, China; ludunke@gzhu.edu.cn

**Keywords:** surface plasmon resonance, refractive index sensor, temperature sensor, optic fiber sensor, two-dimensional materials

## Abstract

Refractive index (RI) and temperature (T) are both critical environmental parameters for environmental monitoring, food production, and medical testing. The paper develops a D-shaped photonic crystal fiber (PCF) sensor to measure RI and T simultaneously. Its cross-sectional structure encompasses a hexagonal-hole lattice, with one hole selectively filled with toluene for temperature sensing. By coating the D-shaped surface of the PCF with a metal film and a MoS_2_ film, the refractive index-detection channel is formed. Numerical results demonstrate that RI and T can be reflected in the same spectrum, without any interference caused by the two parameters with each other. At an environmental RI of 1.26–1.38, its maximum RI sensitivity is up to 5400 nm/RIU. At a temperature of 20–80 °C, its temperature sensitivity reaches −1.2 nm/°C. This design allows for a broad operational spectrum and an extensive measurement range, which makes it particularly suitable for applications requiring low-RI detection. Moreover, the resonance strength of the sensor is significantly enhanced by introducing a two-dimensional material MoS_2_ on the D-surface. Specifically, it reaches 195,149 dB/m when RI = 1.34 at 30 °C. This is much higher than that of most previous studies, and the requirements for inspection equipment can be lowered in this case. These results are essential for progress in simultaneously detecting RI and T.

## 1. Introduction

Temperature (T) and refractive index (RI) measurement crucially affects bio-sensing, environmental monitoring, medical testing, food production, etc. Optical fiber sensors exhibit structural compactness, strong sensitivity, robustness to electromagnetic interference, low cost, and integration feasibility, and thus are crucial for RI and T detection [1,2,3,4]. The existing optical fibers sensors on the market are Optical Fiber Bragg Grating (FBG) Sensors, Optical Fiber SPR (OFS) Sensors, Interferometric Fiber Optic (IFO) Sensors, etc. [5,6,7,8]. Among them, OFS sensors attract more attention with regard to their application in measuring T and RI [9,10,11,12,13]. However, most of these reports focus on the single-channel detection of RI or T. Actually, given that RI and T are closely related in many fields, OFS sensors can assist well in simultaneously detecting the two indexes.

In the past few years, there has been some research conducted on dual-channel sensors for measuring RI and T. Specifically, Feng [14] and Luo et al. [15,16] worked on the simultaneous detection of RI and T based on SPR with a traditional SPR structure, but the structure lacks compactness. In 2017, Jesús Salvador Velázquez-González et al. [17] put forward an OFS sensor for simultaneously detecting RI and T using a MM-SM-MM fiber structure. Its maximum sensitivities reached 2323.4 nm/RIU and −2.850 nm/°C, respectively. In 2021, L. Liu and Zhi Liu et al. developed a SMF SPR sensor to simultaneously measure RI and T [18]. With the RI in the range of 1.333–1.390, the RI sensitivity reached 2260.1 nm/RIU. With the temperature rising from 20 to 60 °C, the corresponding sensitivity was −2.41 nm/°C. PCF, as a special type of optical fiber with a flexible structure, has been widely applied in optical fiber devices [19,20]. In 2022, Teng et al. [21] proposed a double-side polished U-shape plastic optical fiber (POF)-integrating SPR sensor for RI and temperature, which, according to experimental results, could yield an RI sensitivity of 1258 nm/RIU within an RI range of 1.34–1.37, and a temperature sensitivity of −0.596 nm/°C at 30–80 °C. In 2023, Tian et al. [22] proposed a dual-channel sensor through coating an Ag-TiO_2_ layer onto the D-shaped surface of PCF. The highest RI and T sensitivities were 6700 nm/RIU and −21 nm/°C, respectively. However, the measuring ranges of the sensor were only 1.35–1.39 and 32–80 °C. To sum up, the RI measuring ranges in most of the previous studies were higher than 1.33. This is because the resonance curve flattens when the RI is low. It is not easily detectable when the RI is too low. In order to detect substances with lower RI, the resonance effect must be well enhanced. As far as we know, two-dimensional (2D) materials can strengthen the effect on SPR well [23]. In recent years, many studies have been conducted on the application of two-dimensional (2D) materials in single channel sensors, and significant progress has been made in improving their sensitivity [24,25]. In 2024, Aryan Abbaszadeh et al. [26] proposed a PCF sensor based on graphene materials, which provides an average wavelength sensitivity and amplitude sensitivity as high as 4000 nm/RIU^−1^ and 16,905.15 RIU^−1^, respectively, for analytes with RIs ranging from 1.33 to 1.342. Despite some studies on 2D materials based optical fiber SPR sensors and sensitivity enhancement, most of the sensors are single-channel ones. Therefore, an enhanced optical fiber SPR sensor should be designed for dual-parameter measurement.

The paper devises a 2D materials enhanced dual-channel PCF-SPR sensor for simultaneously detecting RI and T. Combining the D-shaped PCF with a simple structure, this sensor comprises three layers of air holes and one polishing plane. Channel-I is formed after the coating of sodium film and MoS_2_ film on the D-surface to detect RI, with toluene selectively filled in one of the second-layer air holes for temperature measurement. The sensor operates within a bandwidth range of 650–1650 nm. As demonstrated, the measurements of the two parameters are independent of each other, thereby reducing both measurement errors and operational complexity. According to the numerical results, this sensor is capable of measuring RI within the range of 1.28–1.38 and temperature within the range of 20–80 °C. Compared with most dual-parameter sensors coated with a single metal, the 2D material coated on the metal film enhances the effect on SPR and widens the measurement range for low-RI materials, as well as preventing metal oxidation as a protective layer. As 2D materials have a high surface area, there is a large surface region for biomolecule adsorption [27]. It is thus very significant to conduct research on dual-parameter optical fiber sensors.

## 2. Structure Design and Sensing Principle

### 2.1. Design of the Sensor Structure

**Figure 1:**
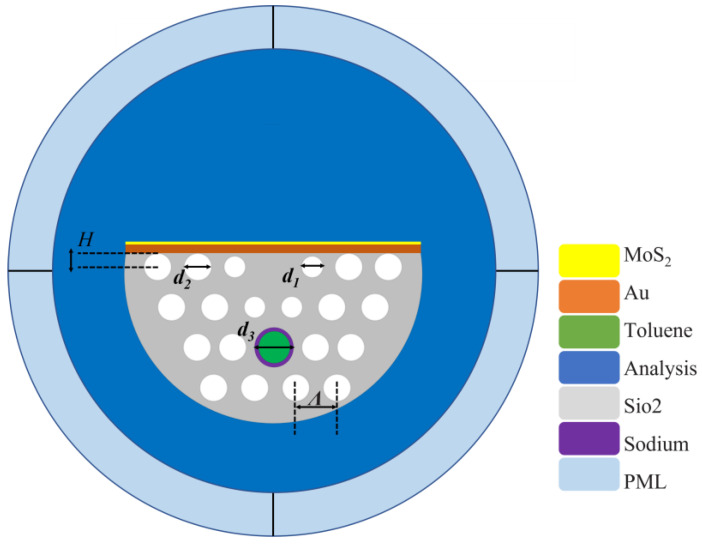
The cross-section of the proposed sensor.

Figure 1 presents the cross-sectional view of the sensor. Based on our knowledge, a 3-layer structure can meet the design requirements for refractive index sensing. Therefore, the PCF encompasses 3 layers of air holes to minimize the complexity of the internal structure. The second layer of air holes was chosen to be filled with temperature-sensitive material. Because the core-guided mode needs to be sensitive to the temperature-sensitive material, it cannot be too far from the core. Meanwhile, to minimize interference with channel-I, the innermost air holes are not a good choice. Additionally, the size of the innermost air holes was appropriately reduced to ensure better transmission of the core mode. This design enhances the overall performance and stability of the sensor. The diameters of differently sized air holes are denoted as *d_1_* and *d_2_*, respectively. *Λ* and *D* represent the lattice pitch between two adjacent air holes and the diameter of the PCF), respectively. A perfectly matched layer (PML), which is positioned outside the outermost ring with an internal diameter of 30 μm and a thickness of 1.5 μm, and scattering boundary conditions were introduced in the calculation process,. The RI of the PML is identical to that of the measured object. The D-surface of the PCF presents the deposition of nano-thickness gold and MoS_2_ films. Between the polishing of the D-surface and the gold film, the core-leak mode and SPP mode excite SPR, forming an RI measurement channel. The surfaces are modified by 8 layers of MoS_2_ for sensor sensitivity enhancement. The sodium film and toluene in one of the air holes assists in the formation of the temperature sensing channel. Table 1 lists the sensor structure parameters in detail.

The sensor’s substrate material is silica, as shown in the gray background of Figure 1. The calculation of the relevant RI is based on the Sellmeier equation, as follows [28]:

(1) $$\left(\lambda \right)=\sqrt{1+\frac{B_{1}\cdot \lambda^{2}}{\lambda^{2}-C_{1}}+\frac{B_{2}\cdot \lambda^{2}}{\lambda^{2}-C_{2}}+\frac{B_{3}\cdot \lambda^{3}}{\lambda^{2}-C_{3}}}$$

In the above equation, λ represents the incident wavelength. The Sellmeier coefficients $B_{1}$ = 0.6961663, $C_{1}$ = 0.00467914826, $B_{2}$ = 0.4079426, $C_{2}$ = 0.0135120631, $B_{3}$ = 0.8974794, and $C_{3}$ = 97.9340025.

The SPR sensor proposed in this study is wavelength-modulated. In some recent studies focusing on SPR sensors, there are many metals used, such as copper, gold, aluminum, silver, etc. Due to the high stability of gold [29], gold film was deposited on the D-surface for the excitation of SPR, forming the Channel-I for RI sensing. Meanwhile, the gold film was covered with 2D materials. Interacting with an evanescent field with 2D materials effectively strengthens the outcome of light absorption. Allowing for this, graphene, MoS_2_, and graphene/MoS_2_ 2D heterojunctions were selected in our design for validation. Finally, MoS_2_ was chosen. In Song et al.’s study [30], the accuracy of the dielectric function of MoS_2_ with different layers was experimentally verified.

As far as we are aware, sensing bandwidth is of much importance, and a wide sensing bandwidth essentially benefits the high performance of dual-channel SPR sensors. To prevent the overlapping of resonance wavelengths between the two channels targeting RI and T detection, different metal films were coated on these sensing channels. In this paper, three different materials, gold, silver, and sodium, were selected for the comparison performed in channel-II. The equation below interprets the dielectric constant of the metal film by virtue of the Drude–Lorentz model [31]:

(2) $$\varepsilon_{m}=\varepsilon_{\infty }-\frac{\omega_{D}^{2}}{\omega \left(\omega +j\gamma_{D}\right)}-\frac{\Delta \varepsilon \Omega_{L}^{2}}{\left(\omega^{2}-\Omega_{L}^{2}\right)+j\Gamma_{L}\omega }$$

where $\varepsilon_{m}$ is the dielectric constant; $\varepsilon_{\infty }$ denotes the high-frequency dielectric constant; Δε indicates a weighting factor; ω refers to the incident light angular frequency; $\omega_{D}$ and $\gamma_{D}$ are referred to as the plasma frequency and damping coefficient, respectively; and $\Omega_{L}$ and $\Gamma_{L}$ indicate the strength of the oscillator and the spectral width of the Lorentz oscillators, respectively. Table 2 lists the parameter values of the Drude–Lorentz model with regard to sodium, gold, and silver.

In addition to the metal film, we also filled an air hole with the thermosensitive material toluene for the formation of channel-II for T sensing. The negative thermal-optical coefficient of toluene reaches −5.273 × 10^−4^ RIU/°C. However, the silica (−6 × 10^−6^ RIU/°C) exhibits a remarkably smaller thermal-optical coefficient versus toluene, and hence we ignore its relevant influence on the calculation. The equation below explains the relationship between the RI of toluene and wavelength [32]:

(3) $$n_{tol}^{2}=\frac{1.17477\lambda^{2}}{\lambda^{2}-0.01825}+1$$

### 2.2. Sensing Principle

**Figure 2:**
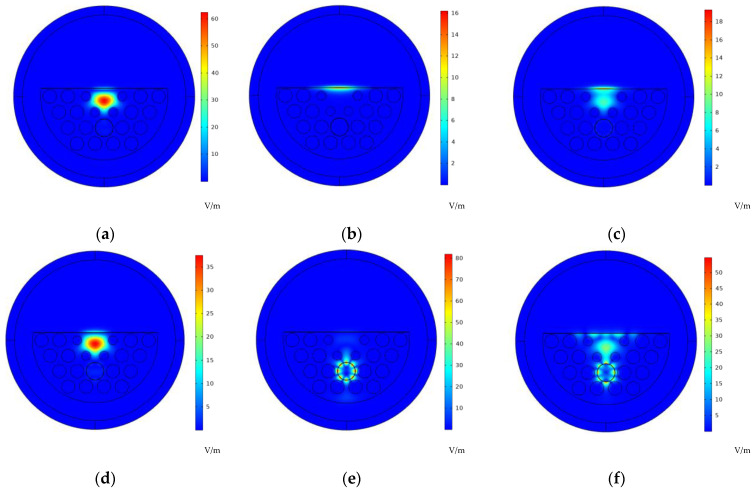
When *n* = 1.34 and *T* = 40 °C, these are the electric field distributions of the (**a**) core mode and (**b**) SPP mode of channel-I at 700 nm, (**d**) core mode and (**e**) SPP mode of channel-II at 900 nm, (**c**) resonant mode of channel-I at 788 nm, and (**f**) resonant mode of channel-II at 1571 nm.

COMSOL Multiphysics 5.4 software was used for model simulation. Table 1 lists the model’s basic parameters. The thicknesses of both the gold film and sodium film was 40 nm, and that of the 8-layer MoS_2_ film was 5.12 nm. Since two different plasma materials were used simultaneously, the proposed sensor divides the evanescent field into many parts. In this way, each material is capable of generating various SPP modes simultaneously. Figure 2 illustrates the light distribution pertaining to the core-guided mode (CGM) and SPP mode. Figure 2a,d show the CGM at 700 nm and 1200 nm, respectively. Obviously, these figures demonstrate the consistent energy transmission in PCF. Figure 2b,e display the SPP modes of the dual channels. As PCF exhibits the dual-channel structure, the CGM leads to the generation of SPR with the gold film on the D-surface and the sodium film in the toluene hole, respectively. Figure 2c,f present the resonance modes corresponding to the two channels with resonant wavelengths of 788 and 1571 nm, respectively. Obviously, energy transmission occurs near the two types of films. The confinement loss (CL) of fiber transmission is a valuable parameter for characterizing SPR properties, expressed as follows:

(4) L(x,y)=40πλln10Im(neff)=8.686×2πλIm(neff)×106

where λ represents the incident light wavelength and Im(neff) represents the imaginary part of the effective RI of the CGM. The CL and wavelength are expressed in dB/m and μm, respectively.

**Figure 3:**
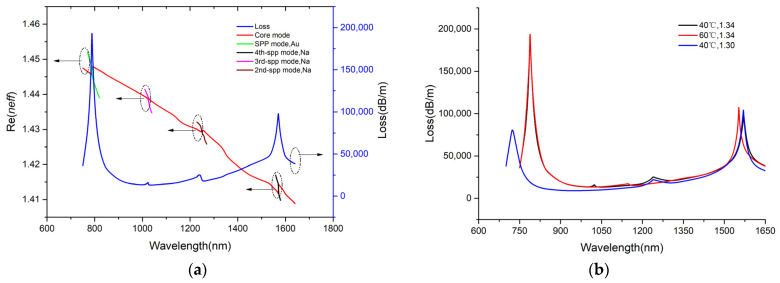
(**a**) Dispersion relationship between core mode and SPP mode of Au film and second, third, and fourth SPP modes of Na film, and loss spectrum at analyte RI of 1.34. (**b**) Loss spectrum of the sensor with different temperatures and RIs.

According to our design, the gold film and the sodium film both stimulate the SPR effect. Allowing for the uniqueness of each metal material, there is a difference in the frequency at which the SPP mode matches the CGM. On that account, some unique phase-matching conditions produce corresponding resonant wavelength peaks. Figure 3a conveys the dispersion relation of the two modes. The CL spectra of the CGM serves as the function of the operating wavelength λ when the RI = 1.34 at 40 °C. As shown in Figure 3a, higher-order SPP modes were generated in channel-II. They caused two small peaks on the resonance curve. These two small peaks did not have a significant impact on the dual-parameter measurement results of the sensor because the resonance intensity between the 2nd and 3rd-order SPP modes and the CGM in channel-II is far lower versus that of channel-I. The CGM and the 4th-order SPP mode have much stronger resonance intensity, and the resonance wavelengths are at the communication waveband. Meanwhile, the SPR peak spacing between the two channels is larger. Evidently, two loss peaks appear in the same spectrum. Figure 3b shows the loss spectrum at varying RIs and temperatures. Peak 1 only shifts with RI, while peak 2 shifts with the T. The changes to the two parameters do not affect each other, which implies the capability of the SPR sensor to detect RI and T within the same spectral range and determine them simultaneously.

## 3. Results and Discussion

### 3.1. Sensing System

**Figure 4:**
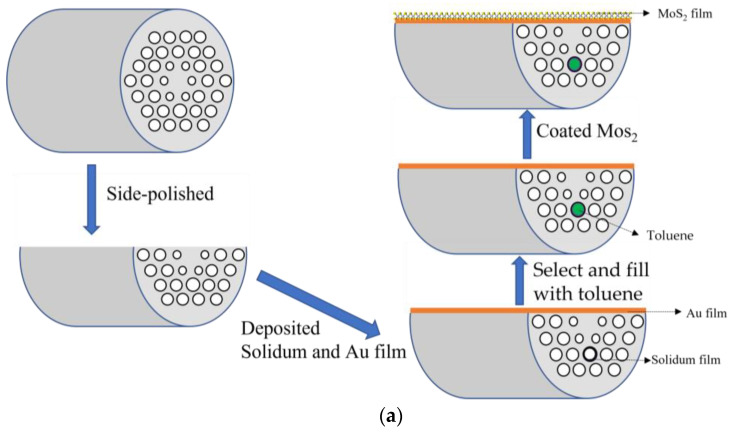

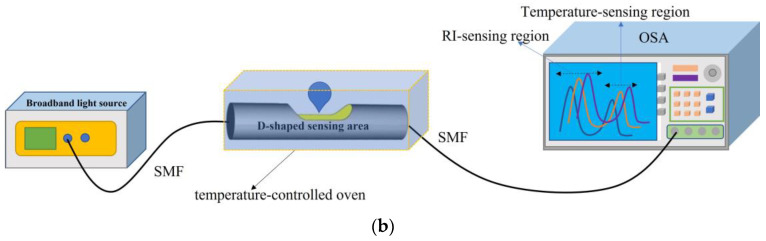
(**a**) The preparation process of the dual-channel sensor, (**b**) a schematic of the transmission-detecting system.

Figure 4a illustrates the relevant fabrication process for our sensor. The PCF designed in this paper is simple in structure, which facilitates its fabrication. We can use the stack and draw method to fabricate the PCF structure with varying diameters for the air holes [33,34]. By arranging and stacking quartz capillaries of different diameters, a preliminary structure of prefabricated rods can be formed. We can then place the stacked capillaries and core rods into a larger quartz tube for packaging. Then, the preform can be placed in a drawing tower and heated to the softening temperature of quartz, making it soft and stretchable. Due to the different sizes of the capillaries, the diameter of the air holes drawn out will also be different. Eventually, PCFs with air holes of different sizes will be formed. The polishing technology, hole filling technology, and coating of the polished surface with a gold film are very mature processes. The toughest challenge lies in the preparation of the sodium thin films. To solve this problem, the solution proposed by Zhang in 2022 can be adopted [35]. They used the liquid-phase deposition (LPD) method to realize a thin silver layer with a thickness of dozens of nanometers coated on the inner surfaces of the air holes to support the excitation of the SPR phenomenon. Subsequently, toluene can be introduced into the air hole. Selective filling enables the introduction of specific materials into designated air holes within the fiber structure. There are several selective-filling methods that can be successfully implemented this process, such as focused ion beam etching [36], microscopic imaging, high-precision mechanical control technology [37], and femtosecond laser drilling technology [38]. In 2019, Wang Yiping et al. proposed a liquid-crystal-filled PCF electro-optic modulator based on selective filling by employing the femtosecond laser-assisted selective infiltration technique [39]. In our design, we can also use femtosecond laser drilling technology. Finally, MoS_2_ can be deposited onto the D-surface through chemical vapor deposition [40]. Figure 4b displays the transmission-detecting system. A broadband incident light is transmitted through SMF to the D-shaped sensing probe, and a temperature-controlled chamber is adopted for temperature controlling. Along with the changing temperature or RI of the analyte, the loss spectrum undergoes a blue shift or red shift. A spectrum analyzer (OSA) is used to detect modulated spectra. In the test sensing system, the sensor designed in this paper is connected to the system by fusion with single-mode fiber. In this design, the difference in structural dimensions between SMF and PCF leads to a significant mode field mismatch, which becomes a major source of coupling loss. To mitigate these losses, various solutions have been proposed [41,42]. However, the splicing performance of optical fibers with substantial differences in structure and mode field diameter remains suboptimal. In 2015, Li Xuyou et al. introduced a slow-tapered PCF interface [43]. By integrating a universal slow-tapered PCF interface between different types of fibers, it is possible to effectively reduce coupling loss, making it an advantageous choice for our system. Furthermore, employing three-dimensional (3D) X-ray imaging can help minimize mechanical alignment errors, ultimately ensuring that the transmission requirements of the system are satisfactorily met.

### 3.2. Analysis of Sensing Performance of Channel-I

#### 3.2.1. Analysis of Sensing Performance of Varying Metal Films of D-Surface

**Figure 5:**
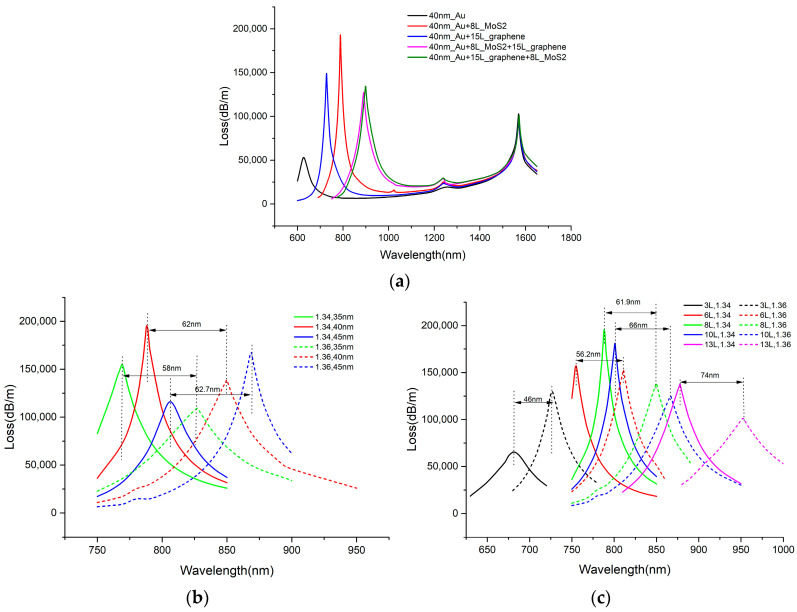
The loss spectra of the core mode of channel-I with different (**a**) 2D materials, (**b**) gold film thicknesses, and (**c**) layers of MoS_2_ film.

As mentioned previously, the two sensing channels present an independence, supporting the separate analysis of their sensing characteristics. Firstly, we explore the sensing characteristics of channel-I for RI detection, confirming the metal film thickness on the D-surface and the selection of 2D materials. Figure 5a presents the fiber CGM loss spectrum captured when the D-surface gold film is covered with 2D materials or not, along with the comparison performed when different 2D materials are used to cover it. The gold film was 40 nm thick, with an RI = 1.34 at 40 °C. The solid black line in the figure represents the loss curve with only gold film coated on the D-surface, while the other curves represent the results obtained after coating 2D materials on the gold film. Evidently, the SPR peak value of the loss remarkably increases upon the coating of the 2D material on the gold film. According to such design, the optical fiber has a CL of roughly 50,000 dB/m when only gold film exists on the D-surface. However, when the 2D material is added to the gold film, the CL increases to approximately 150,000 dB/m, which is three times higher than when there was only a gold film, indicating that 2D materials enhance the SPR effect. Therefore, it is considered a sensible choice to optimize the sensing characteristics by using 2D materials to cover the gold film. Figure 5a also shows the influence of different 2D materials on the transmission characteristics of the sensor, including MoS_2_ and graphene, alongside their heterojunctions. When only the 40 nm thick MoS_2_ is covered, the resonance intensity reaches the maximum level and the resonance peak becomes sharper, which is indicative of the strong capability of the sensor to more accurately measure the presence of or changes in target substances. Therefore, it is inferred that the best option for this design is to cover the gold film surface with the MoS_2_ material. Next, a further discussion will be conducted on how the sensing characteristics are affected by the gold film’s and the MoS_2_ film’s thickness.

#### 3.2.2. Selection of Gold Film Thickness on the D-Surfaces

As indicated by the solid lines in Figure 5b, the SPR peak exhibits a red shift with rising gold film thickness. When the RI reaches 1.34, the resonance wavelengths at the gold film thicknesses of 35, 40, and 45 nm are 769.0, 788.0, and 806.3 nm, respectively. Also, the resonance intensity changes with the gold film thickness, reaching a maximum of 195,618 dB/m at a gold film thickness of 40nm. At the same time, the resonance peaks all shift towards longer wavelengths as the RI rises. When tgold = 35 nm or 40 nm, the resonance intensity declines as the RI rises; when tgold rises to 45 nm, it shows an increasing trend. At three different metal film thicknesses of 35, 40, and 45 nm, the sensor presents a wavelength sensitivity of 2900, 3100, and 3700 nm/RIU, respectively, along with a slight change in RI from 1.34 to 1.36. The gold thicknesses proved to slightly affect the sensitivity. Given its maximum resonance strength, we set the gold film thickness to 40 nm. When tgold = 40 nm, the loss reaches 195,000 dB/m, so we chose 40 nm as the gold film thickness for this design.

#### 3.2.3. Selection of MoS_2_ Film Thickness on the D-Surfaces

Figure 5c illustrates the fiber CL in the case of the MoS_2_ film thickness altering from 3 layers to 13 layers at two different RI values of 1.34 and 1.36. Obviously, the thickness of the MoS_2_ dramatically affects the working band of channel-I. With rising MoS_2_ thickness, the SPR peak shifts towards the longer wavelength band. When RI = 1.34, the resonance intensity rises sharply as the thickness of MoS_2_ increases from three layers to eight layers. However, when the thickness increases to 13 layers, the resonance strength declines. The rising in the liquid RI causes a shift in the resonance wavelength toward the long band. When the thickness is 3, 6, 8, 10, or 13 layers, the wavelength sensitivity reaches 2300, 2810, 3095, 3300, and 3700 nm/RIU, respectively. Evidently, thicker MoS_2_ indicates higher wavelength sensitivity. However, it is also noteworthy that when the thickness of MoS_2_ is either 3 or 13 layers, the resonance curve becomes flatter, which restricts the measuring range of the sensor. When the thickness of MoS_2_ is 6 layers, the resonance intensity is relatively stable with the change in RI, but the sensitivity is lower than that of 8 and 10 layers. As the thickness rises to 10 layers, the sensitivity reaches a higher level than that of 6 and 8 layers, but the resonance intensity reaches a lower level than that of 8 layers. Considering the sensor measuring range and sensitivity, the thickness of MoS_2_ was set to eight layers for this design.

### 3.3. Analysis of Sensing Performance of Channel-II

To a large extent, the T-sensing channel is affected by the temperature-sensitive material and metal material of the air hole. Therefore, this section explores the impact of the thermal sensitive material, metal material, and metal layer thickness on the sensing performance.

#### 3.3.1. Selection of Thermally Sensitive Materials to Fill the Air Hole

**Figure 6:**
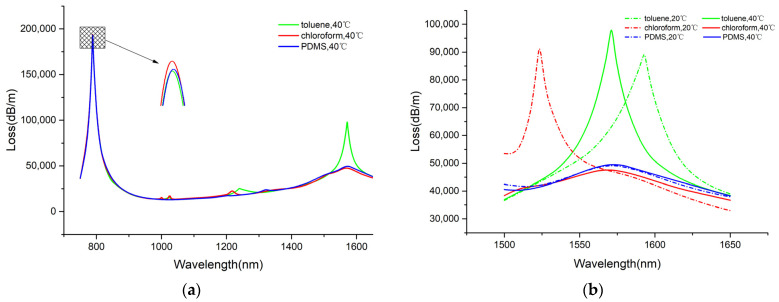
The loss spectra of the core mode with different (**a**) thermally sensitive materials and (**b**) temperatures.

Thermosensitive materials are crucial for temperature sensing in channel-II, because the RI of temperature-sensitive materials is significantly affected by temperature, which causes the SPR peak to shift with temperature. By selecting appropriate thermosensitive materials, the performance and functionality of sensors can be improved effectively. Next, a discussion will be conducted about the impact of different temperature-sensitive materials on the dual-channel sensor designed in this paper. The curve in Figure 6a reflects the impact of thermal sensitive materials on the sensing characteristics at a temperature of 40 °C and an RI of 1.34. The thermal-sensitive material has a significant influence on the temperature-sensing characteristics of channel-II. However, the SPR peak of channel-I is unaffected by the thermally sensitive materials, which implies that the thermally sensitive materials do not affect the sensing performance of channel-I. It is observable that the case of a channel filled with toluene guarantees a sharper resonance peak for channel-II and a higher resonance intensity. Next, an analysis was conducted regarding the way thermosensitive materials affect the sensor performance of channel-II at different temperatures. According to Figure 6b, when the air hole is filled with toluene, distinct resonance peaks emerge at different temperatures. When the channel is filled with chloroform, the loss peak becomes extremely flat at 40 °C. When it is filled with PDMS, the loss spectrum of channel-II is very flat at 20 and 40 °C, with the resonant peak position and magnitude remaining unchanged basically. In conclusion, toluene is the optimal choice for temperature sensitive materials in this design.

#### 3.3.2. Selection of the Metal Film for the Air Hole

**Figure 7:**
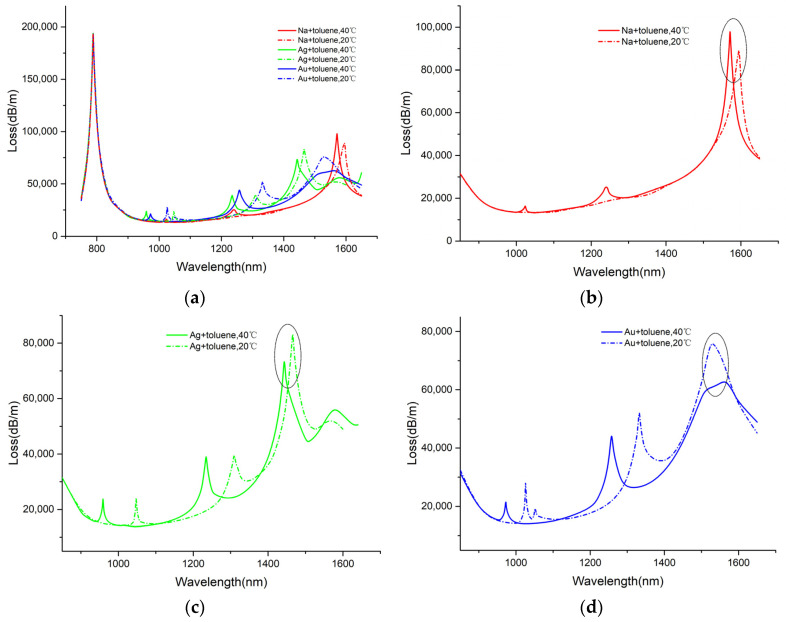
The loss spectra of the core mode with (**a**) different metal materials in channel-I and channel-II, (**b**) sodium film in channel-II, (**c**) silver film in channel-II, and (**d**) gold film in channel-II. The circles marked in figures (**b**–**d**) are the resonance peaks selected for temperature measurement in this design.

In channel-II, the air hole is not only filled with toluene but also coated with metal film inside. The metal film material importantly affects the SPR effect and SPR sensor performance. Choosing the appropriate metal material for channel-II is also important work. Therefore, this part of the work analyzes the influence of metal materials on the sensing performance of channel-II. Figure 7a presents the CGM loss spectra upon the coating of gold, silver, and sodium on the air hole. The temperature at 20–40 °C leads to the shift in the resonance peak of channel-II toward the short waveband, while that of the channel-I is still at 788 nm. The resonance peaks of channel-I do not shift when the metal film coating the air hole varies, demonstrating the independence between the two sensor channels during measurement. Thus, the strong independence and stability of the two sensing channels are confirmed, together with remarkably reduced measurement errors and complexity. Furthermore, the influence of the metal material on channel-II was analyzed. Figure 7b–d display the temperature-sensing performance of the air hole when coated with gold, silver, and sodium, respectively, at a film thickness of 40 nm. As revealed by comparing the three simulation results, there are multiple resonance peaks appearing in the 800–1600 nm wavelength range when silver film and gold film coatings are used, because of the CGM coupling with the higher-order SPP mode. It is also notable that the wavelength of some resonance peaks is closer to that of channel-I. Especially in the short-waveband, the wave peaks of RI and temperature may overlap, which is adverse to the operation of dual-channel sensors. Comparatively, the interference loss peak of the loss spectrum based on sodium film is much weaker, with almost no excessive resonance peaks exhibited when T = 20 °C. It can also be found out that the resonance intensity of channel-II is relatively lower compared to channel-I. Allowing for resonance intensity, it is preferable to choose a peak with a high resonance intensity as the detection peak for channel-II. Since the resonance intensity of the long-waveband SPR resonance peak is higher than other peaks, the peaks marked in Figure 7b–d can be treated as the detection peak for channel-II. Obviously, the sodium-coated SPR sensor had a larger resonance intensity and a larger sensing bandwidth than the those coated with silver or gold. Hence, we selected the sodium film as the metal for the excitation of the SPR effect for channel-II.

#### 3.3.3. Selection of Sodium Film Thickness

**Figure 8:**
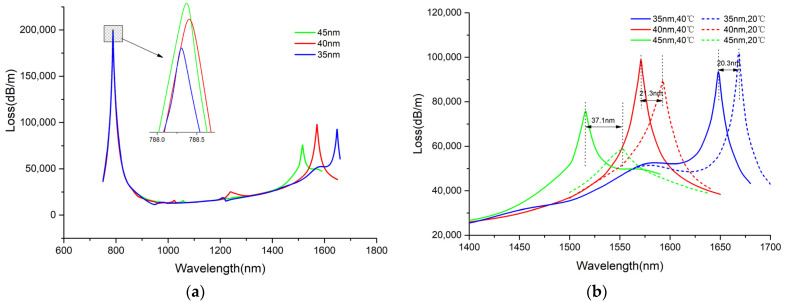
The loss spectra of the core mode with different (**a**) sodium film thicknesses when T = 40 °C, and (**b**) temperatures.

As far as we know, the variations in metal film thickness can affect the matching conditions of the SPR. In this study, it is investigated how the sodium film thickness affects the temperature-sensing channel. Figure 8a presents the SPR peaks in channel-II undergoing a blue shift as the sodium film thickness rises from 35 to 45 nm at 40 °C. The resonance wavelength changes from 1648 to 1515 nm. Notably, the resonance intensity reaches its maximum when the sodium film is 40 nm thick. For channel-I, neither the resonance wavelength nor the resonance intensity was affected by changes in sodium film thickness, which substantiates a strong independence between the two channels. Furthermore, the resonance wavelength undergoes a red shift as the temperature decreases. For instance, in the case of a 40 nm-thick sodium film, the resonance wavelength shifts from 1571 to 1593 nm as the temperature drops from 40 to 20 °C (Figure 8b). When the sodium film is 45 nm thick, the resonance strength is the lowest, particularly at 20 °C. Under these conditions, the SPR curve becomes notably flatter, suggesting that a thickness of 45 nm is not preferable. With a 35 nm thick sodium film, the SPR peak is sharper than at 40 nm. However, it is also observed that a thicker sodium film results in a lower sensitivity. However, a thicker sodium film is associated with a higher wavelength sensitivity, as shown in Figure 8b. Considering both sensitivity and SPR spectroscopy, the sodium film thickness was set to 40 nm.

## 4. Performance Analysis of Dual-Channel Sensing

**Figure 9:**
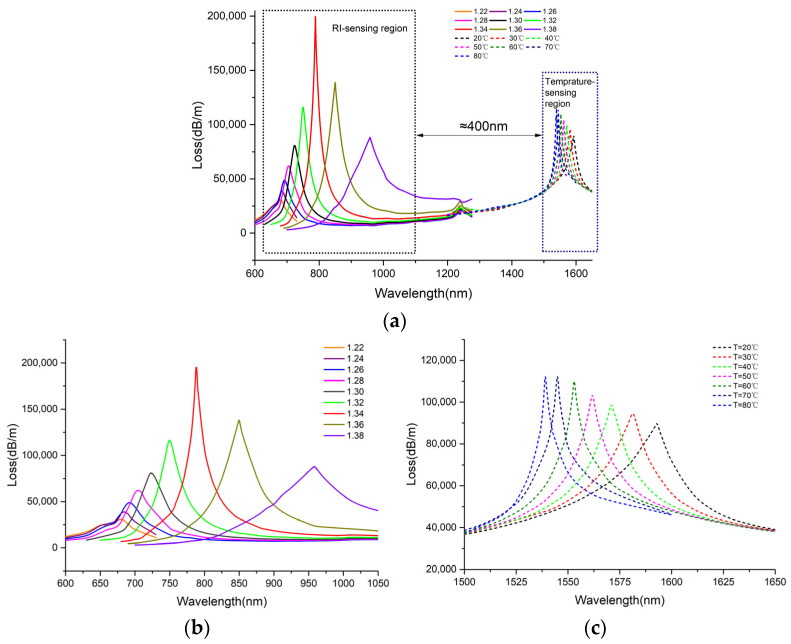
Loss spectra of the designed sensor. (**a**) Changes in the loss spectra with different liquid RIs and temperatures; (**b**) when RI increases from 1.22 to 1.38 with steps of 0.02 and when T = 40 °C; (**c**) when temperature increases from 20 to 80 °C with steps of 10 °C and when RI = 1.34.

Based on the previous analyses, the parameters of the dual channel sensor were determined. Furthermore, its overall performance was evaluated. Figure 9a presents the loss spectra of the fiber CGM for various RIs and temperatures as the wavelength varies from 600 to 1650 nm. Within the 600 to 1250 nm wavelength range, the SPR peak position shifts in response to changes in the RI, establishing the RI sensing region. Analogously, the temperature sensing region is located around 1550 nm, where the sensor exhibits a significant response to T variations. Obviously, the two sensing areas differ greatly, their gap reaching about 400 nm. This prevents the resonance wavelength overlapping for the two sensing channels. Then, a numerical analysis of the two sensing regions was conducted. Sensitivity, resolution, and the figure of merit (FOM) were employed to indicate sensor performance. The changes in RI and temperature were accompanied by the shift in the resonance wavelength, hence, measuring the resonance wavelength shifts could assist in analyzing the fiber RI sensitivity and temperature sensitivity. The wavelength interrogation approach was adopted for confirming the sensor sensitivity. The wavelength sensitivity $S_{\lambda }$ of RI and temperature can be derived from Equations (5) and (6):

(5) $$S_{\lambda }=\frac{\Delta \lambda_{peak}}{\Delta n}\;(\mathrm{nm}/\mathrm{RIU})$$

(6) $$S_{\lambda }=\frac{\Delta \lambda_{peak}}{\Delta T}\;(\mathrm{nm}/{}^{\circ}C)$$

where $\Delta \lambda_{peak}$ represents the resonant wavelength shift, while Δn and ΔT represent the variation in RI and the temperature.

The resolution of sensor is expressed as follows:

(7) $$R=\frac{\Delta n\times \Delta \lambda_{\min}}{\Delta \lambda_{peak}}\;(\mathrm{RIU})$$

where $\Delta \lambda_{min}$ represents the minimum spectral resolution. In our analysis, $\Delta \lambda_{min}$ was set to 0.1 nm.

The FOM is defined as:

(8) $$FOM=\frac{S}{FWHM}\;({\mathrm{RIU}}^{-1})$$

where FWHM is the full width at half the max resonance.

Figure 9b,c present the enlarged view of the RI- and temperature-sensing regions in Figure 9a, respectively. Since the resonance intensity falls below 50,000 dB/m and resonance wavelength presents the minimal change when the RI is 1.22 or 1.23, it is inappropriate to use the resonance wavelength detection method when the RI is lower than 1.23. Therefore, the measuring range was set from 1.26 to 1.38 for RI. According to Figure 9b, Δλ and Δn were obtained. Also, Equations (5) and (7) were used to calculate the RI sensitivity and resolution. Table 3 lists the calculation results. The max sensitivity was 5425 nm/RIU at an RI of 1.36. As the liquid RI rises from 1.26 to 1.34, FWHM gradually decreases. However, as the RI rises from 1.34 to 1.38, FWHM gradually increases. When the RI is 1.34, it leads to a minimum FWHM of 25.74 nm and a max FOM of 120.43 RIU^−1^, as calculated by Equation (8).

Similarly, Equations (6) and (7) were used to calculate the temperature sensitivity and resolution. The calculation results are presented in Table 4. When the liquid temperature is 40 °C, the max wavelength sensitivity is 1.2 nm/°C. When the temperature is 60 °C, the sensor achieves its max FOM, with an FWHM of 16.67 nm and an FOM of 0.048 °C^−1^.

**Figure 10:**
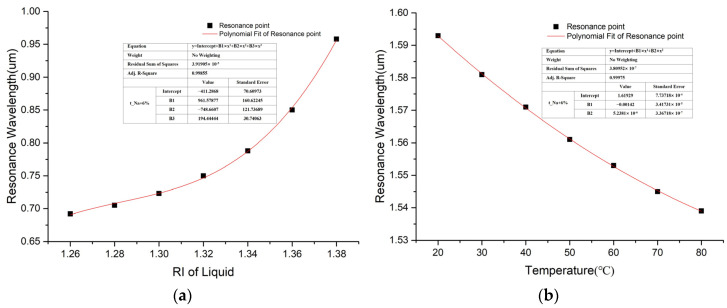
Fitting results between resonance wavelength and (**a**) RI and (**b**) temperature.

Figure 10a,b present the relationship-fitting result between the resonant wavelength and the RI and the temperature of the liquid, respectively. In Figure 10a, the adjusted R-square (R^2^_adj) value is 0.99855, and the RI ranges from 1.26 to 1.38. Substances with RI < 1.33 are detected. In Figure 10b, the R^2^_adj value is 0.99975, hinting a better fit goodness. Compared with previous research, the operating wavelength of channel-II in this paper is near the 1550 nm communication band, which allows for the expanded application fields of dual-channel sensors. Moreover, the two sensing channels operate at significantly different wavelengths, which leads to minimal crosstalk between them. Table 5 details the comparative analysis of the performance of the SPR-based fiber sensor in simultaneously measuring RI and temperature versus other works. Despite no significant improvement in sensor sensitivity, the measuring range of the sensor is widened, especially for RI sensing.

**Figure 11:**
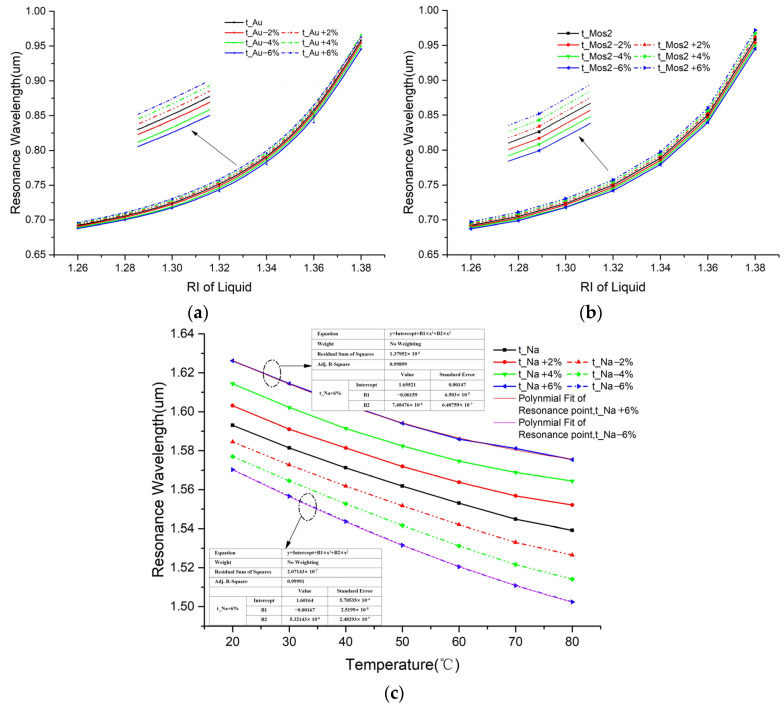
Impact of (**a**) metal film thickness and (**b**) MoS_2_ film thickness tolerance on channel-I, and (**c**) sodium film thickness tolerance on channel-II.

Finally, we analyzed the preparation tolerance of the proposed sensor. In our design, the metal film and MoS_2_ film are important parameters that affect the performance of the sensor. Therefore, we discuss the geometric error of Au film, MoS_2_, and sodium film. We deduced that the parameter of thickness can vary by ±2%, ±4%, and ±6% based on the optimal conditions found. As shown in Figure 11a,b, we calculated the resonance wavelength for channel-I with the tolerance of Au film and MoS_2_ film. With the change in membrane thickness, the trend of the resonant wavelength of channel-I is consistent with the previous research. When the tolerance of the gold film is within ±2%, ±4%, and ±6%, the maximum wavelength shifts within the measurement range are 4.3, 8.4 and 12.9 nm, respectively, when RI = 1.38. The tolerance results of MoS_2_ film are 4.5, 8.9, and 13.5 nm. We can choose to control the tolerance within ±4% for channel-I, which would keep the RI-measurement error within 10%. Figure 11c shows the results for channel-II with the tolerance of sodium film. The tolerance results of sodium film are 12.9, 25.2, and 36.8 nm when T = 80 °C. Although channel-II is greatly affected by the sodium film, regardless of the tolerance range, the temperature exhibits a good binomial relationship with the resonance (as shown in the fitting relationship with a tolerance of ±6%). In practical use, the temperature can still be accurately measured after calibration processing. In addition, even with a tolerance range of ±6%, the two sensing regions remain sufficiently far apart, ensuring that they do not interfere with each other. It is assumed that the proposed PCF has good tolerance to fabrication errors.

## 5. Conclusions

This paper focuses on proposing a dual-channel SPR-PCF sensor to simultaneously detect RI and T in an ultra-wide spectral range. RI is measured by channel-I, deposited with 40 nm gold film and modified by 8 layers of MoS_2_. In contrast, channel-II is coated with 40 nm sodium film and filled with toluene for temperature measurement. According to the study results, the max RI sensitivity is 5400 nm/ RIU at an RI of 1.26 to 1.38, while the FOM for RI sensing is 120.43 RIU^−1^. At a temperature of 20 to 80 °C, the max sensitivity is −1.2 nm/°C and the FOM is 0.048 RIU^−1^. Versus other SPR dual-channel sensors, the sensor proposed here has the following advantages: (1) Extensive measurement range. The RI measurement range is wider than most previous studies, especially for those with an RI lower than 1.33. (2) The temperature working wavelength of most previous dual-channel sensors is around 1200 nm, while it is at the communication wavelength of 1550 nm in this design. As a result, the working wavelength of the temperature measurement is further away from that of the RI measurement, ensuring that there is no spectral overlap within the maximum measurement range of the two parameters. This research is significant for the development of broadband dual-channel sensing, allowing for widespread application in biosensing, environmental monitoring, medical testing, and food production.

## Figures and Tables

**Table 1:** Sensor design parameters.

Parameter	Symbol	Value
Air hole spacing	*Λ*	3 μm
Air hole diameter	*d_1_*	1.5 μm
Air hole diameter	*d_2_*	2.25 μm
Air hole diameter	*d_3_*	3 μm
Polishing depth	*H*	1.3 μm
Diameter of PCF	*D*	21 μm

**Table 2:** Parameter of sodium, gold, and silver.

Symbol	$\varepsilon_{\infty }$	Δε	$\Gamma_{L}/2\pi$	$\omega_{D}/2\pi$	$\gamma_{D}/2\pi$	$\Omega_{L}/2\pi$
Au	5.9673	1.09	104.86	2113.6	15.92	650.07
Ag	2.4064	1.6604	620.7	2214.6	4.8	1330.1
Na	0.500	0.280	4111.38	8225.31	15.19	4487.91

**Table 3:** Performance of the designed SPR sensor for RI.

Analyte (na)	Shift of Δna	Peak Loss (dB/cm)	Reson. Peak Wave *λ_peak_* (nm)	Reson. Peak Shift Δ*λ_peak_* (nm)	Wave. Sens. *S_λ_* (nm/RIU)	Resolution R (RIU)	FWHM (nm)	FOM (RIU^−1^)
1.26	0.02	488.19	692	13	650	1.538 × 10^−4^	45.92	14.15
1.28	0.02	618.86	705	18	900	1.111 × 10^−4^	45.35	19.85
1.30	0.02	806.48	723	27	1350	7.407 × 10^−5^	44.78	30.15
1.32	0.02	1162.60	750	38	1900	5.263 × 10^−5^	39.11	45.58
1.34	0.02	1951.49	788	62	3100	5.263 × 10^−5^	25.74	120.43
1.36	0.02	1388.85	850	108	5400	3.226 × 10^−5^	53.14	101.62
1.38	0.02	879.07	958	-	-	-	-	-

**Table 4:** Performance of the designed SPR sensor for temperature.

Temperature (T)	Shift of ΔT	Peak Loss (dB/cm)	Reson. Peak Wave *λ_peak_* (nm)	Reson. Peak Shift Δ*λ_peak_* (nm)	Wave. Sens. *S_λ_* (nm/°C)	Resolution R (RIU)	FWHM (nm)	FOM (°C^−1^)
20	10	897.70	1593	12	1.2	0.0833	32.33	0.037
30	10	943.07	1581	10	1.0	0.1	27.33	0.036
40	10	988.79	1571	10	1.0	0.1	23.81	0.042
50	10	1032.15	1561	8	0.8	0.125	20.78	0.038
60	10	1100.93	1553	8	0.8	0.125	16.67	0.048
70	10	1121.75	1545	6	0.6	0.167	13.51	0.044
80	10	1119.63	1539	-	-			

**Table 5:** A comparison of the results of the dual channels of previous sensors and the proposed sensor.

Reference	Structure	RI				Temperature			
		RI Range (RIU)	Max Sensitivity (nm/RIU)	FOM (RIU^−1^)	Resolution (RIU)	T Range (°C)	Max Sensitivity (nm/°C)	FOM (°C^−1^)	Resolution (RIU)
[17]	MM-SM-MM (2017)	1.33–1.38	2323.4	-	5 × 10^−5^	20–60	−2.85	-	-
[44]	D-shaped PCF (2021)	1.33–1.34	1371	-	-	0–40	−1.06	-	-
[45]	Cascade structure fiber (2023)	1.34–1.38	3820	83.77	-	20–80	−5.189	0.054	-
[46]	Two single-D-shape PCF (2023)	1.30–1.40	1.2500	24.75	8 × 10^−6^	−60–100	0.7	13.37	0.0142
[47]	D-shaped open-ring PCF (2023)	1.33–1.40	7700	-	4.17 × 10^−4^	0–60	6.1	0.235	0.00163
[48]	Glass fiber core (2024)	1.33–1.38	4774	36.07	6.28 × 10^−5^	20–90	1.76	-	-
Proposed work	D-shaped PCF	1.26–1.38	5400	120.43	7.407 × 10^−5^	20–80	−1.2	0.048	0.167

## Data Availability

The original contributions presented in this study are included in the article. Further inquiries can be directed to the corresponding author.

## Abbreviations

The following abbreviations are used in this manuscript:

PCF	Photonic crystal fiber
CGM	Core-guided mode
RI	Refractive index
CL	Confinement loss
T	Temperature
FOM	Figure of merit
